# Identifying novel indicators of non-technical skills derived from operative video annotation

**DOI:** 10.1093/bjs/znag015

**Published:** 2026-02-19

**Authors:** Lachlan Dick, Connor Boyle, Victoria Ruth Tallentire, Joel Norton, Emma Howie, Douglas S Smink, Richard J E Skipworth, Steven Yule, the Surgical Video Grading and Assessment (SVGA) Group, Mohamed Abdulmajed, Kamal Aryal, Ajay P Belgaumkar, Daniel Beral, Paul M Brennan, Euan Bright, Leo R Brown, Stuart Clark, Sara Downey, Jeremy French, Ewen A Griffiths, Leo McCormick Matthews, M J Proctor, Ahmed Saad, Lasitha B Samarakoon, Tim Stansfield, Graham Sunderland, John V Taylor, James Tomlinson, John Wayman, J A Young

**Affiliations:** Surgical Sabermetrics Laboratory, Usher Institute, University of Edinburgh, Edinburgh, UK; Medical Education Directorate, Royal Infirmary of Edinburgh, NHS Lothian, Edinburgh, UK; Clinical Surgery, University of Edinburgh, Edinburgh, UK; Surgical Sabermetrics Laboratory, Usher Institute, University of Edinburgh, Edinburgh, UK; Clinical Surgery, University of Edinburgh, Edinburgh, UK; Medical Education Directorate, Royal Infirmary of Edinburgh, NHS Lothian, Edinburgh, UK; Surgical Sabermetrics Laboratory, Usher Institute, University of Edinburgh, Edinburgh, UK; Clinical Surgery, University of Edinburgh, Edinburgh, UK; Surgical Sabermetrics Laboratory, Usher Institute, University of Edinburgh, Edinburgh, UK; Clinical Surgery, University of Edinburgh, Edinburgh, UK; Department of Surgery, Brigham and Women’s Hospital/Harvard Medical School, Boston, Massachusetts, USA; Surgical Sabermetrics Laboratory, Usher Institute, University of Edinburgh, Edinburgh, UK; Clinical Surgery, University of Edinburgh, Edinburgh, UK; Surgical Sabermetrics Laboratory, Usher Institute, University of Edinburgh, Edinburgh, UK; Clinical Surgery, University of Edinburgh, Edinburgh, UK

## Abstract

**Background:**

Cognitive non-technical skills (NTS), including situation awareness and decision-making, are critical determinants of surgical outcomes. Current NTS assessments depend on expert human observation, which is resource-intensive and difficult to scale. To address this, we investigated whether surgical gestures, derived from annotated video of the surgical field, could serve as objective indicators of cognitive NTS.

**Methods:**

A data set of 40 open-source laparoscopic appendicectomy videos was annotated for temporal (for example surgical gestures) and spatial (for example coordinates of actions) indicators of surgical NTS. Using the Non-Technical Skills for Surgeons (NOTSS) tool, 12 expert observers independently assessed decision-making (1–4) and situation awareness (1–4). Multivariable linear regression analysed video-derived indicators predictive of NTS.

**Results:**

Across all videos, a total of 10 385 events were annotated, generating 87 374 data points. The mean cognitive NOTSS rating was 5.6 (s.d. 0.8) out of 8, with decision-making and situation awareness highly correlated (r = 0.8, *P*  *<*  *0.001*). The final multivariable model explained 39.6% of the variance in expert cognitive NOTSS ratings, identifying five predictors: dexterity index, events during the final operative phase, coagulating events, dropping actions, and actions targeting the small bowel.

**Conclusion:**

This study provides evidence that non-technical skills can be inferred from silent video of the surgical field alone. These findings lay the foundations for scalable, automated tools to evaluate surgeons’ cognitive processes offering new avenues to improve surgical training, performance and outcomes.

## Teaser

Developing data-driven insights into cognitive non-technical skills would offer an alternative to resource-intensive expert observation. Using annotated laparoscopic appendectomy videos, gesture-based metrics accounted for a substantial proportion of variance in expert ratings, with specific temporal features emerging as key predictors. These findings support the feasibility of scalable, automated assessment tools to inform training and enhance operative performance.

## Introduction

The field of surgery remains one of the highest-risk arenas within modern healthcare. As the third leading contributor to global deaths, improving surgical standards is an urgent priority^1^. Although variation in postoperative outcomes is influenced by many factors, recent attention has focused not only on patient characteristics but also on the surgeon’s non-technical skills (NTS). These critical cognitive (for example decision-making) and social (for example leadership) domains in the operating room (OR) are essential for safe and effective care and are increasingly becoming embedded into surgical training pathways^2^. A recent systematic review including over 250 000 procedures established a significant relationship between intraoperative NTS and patient-level outcomes^3^. Perioperative NTS (for example consideration of treatment options) also influence outcomes, particularly in high-risk patients, reinforcing the need for regular appraisal and feedback^4^. This is especially relevant for situation awareness and decision-making given their greater representation in NTS errors^5^. Current approaches for assessing these higher-order cognitive skills predominantly rely on expert surgeons. Regular NTS feedback is not commonplace and, when performed, is time intensive. Debriefing a single procedure can take a median of 30 min, which can translate into several hours for a whole list^6^. Developing automated metrics could improve scalability of routine NTS appraisal by highlighting debriefing focus and quality of feedback.

The rapid evolution of operative video analysis offers unprecedented opportunities for novel, enhanced performance insights^7^. The potential for evaluating NTS through data was identified in a recent international Delphi, in which NTS-based operative performance metrics represented the most prominent domain (4 of 10 statements) within the consensus statement^8^. Feasibility of deep-learning to assess operating team NTS has previously been demonstrated using wide-angle video of the whole OR^9^; however, there are currently no approaches to analyse NTS from video of the surgical field alone^10^. Given the ubiquity of minimally invasive surgical video and the urgent need for scalable objective feedback, developing tools to measure cognitive NTS using existing operative video feeds and without the need for expert assessors would represent a significant advance in surgical performance assessment and quality improvement.

Surgical gestures, defined as the classification of specific actions (for example cutting), are currently used for competency and quality assessment of technical skills^11^. Gestures can be further characterized by action triplets—an action, the target of the action (for example skin), and the instrument used (for example scalpel)^12^. Specific gesture use, determined through video annotation, has been shown to directly impact functional outcomes following surgery, further strengthening their potential to provide meaningful insights for surgeons and influence patient care^13^. Although video-derived gesture analysis has successfully captured technical skills, its application to assess NTS remains unexplored. The potential to harness surgical gestures to reflect cognitive abilities is strengthened by the increasingly established relationship between technical and NTS^14^. For example, repetition of movements and variations in surgical workflow could be used to reflect decision-making processes.

To advance the field of surgical data science, the present study explored to what extent surgeons’ gestures, derived from surgical field video, could serve as objective markers of cognitive NTS and how expert raters infer such skills when broader operative context is unavailable. Establishing such relationships is a necessary precursor to automation, validation, and eventual integration into surgical training and quality improvement frameworks.

## Methods

This study leveraged expert-ratings and annotation of operative video to identify attributes from the surgical field to represent situation awareness and decision-making. Laparoscopic appendectomy was chosen given its prevalence, with over 300 000 procedures performed annually in the United States, most commonly by surgical trainees, with postoperative complications reported in up to 13% of cases^15–17^. Approval for this study was granted by the Edinburgh Medical School Research Ethics Committee (reference: 24-EMREC-020_A1).

### Data set development

A data set of 40 open-source laparoscopic appendectomy videos was developed by searching YouTube (California, USA) for ‘laparoscopic appendectomy’ and ‘laparoscopic appendicectomy’. Videos were eligible if they were unedited and demonstrated a non-retrocecal, uncomplicated appendicitis, defined as grade 1 or 2 on the acute appendicitis disease severity score (*Table S1*)^18^. Two independent raters (L.D., C.B.), each with >100 appendectomies performed as the primary surgeon, assessed 10 videos (25% of the data set) for disease severity and interrater reliability was calculated. To further standardize the data set, included videos had to ligate the appendiceal base with at least one surgical tie without the use of a stapling device. This operative technique was chosen as it allowed further demonstration of cognitive processes (for example placement of the surgical tie) and is a frequently used approach for appendiceal base ligation^19^. Otherwise, instrument selection was at the discretion of the operating surgeon.

Each video was edited to show a 5-min segment immediately before the first surgical tie on the appendix was cut. This allowed for both surgical tie application and dissection skills to be included, providing raters with a broad range of skills to assess. Previous studies utilizing video-based ratings have adopted a similar method showing only critical steps to represent the whole procedure^20^.

### Cognitive NTS evaluation

Video segments were rated by members of the Non-Technical Skills for Surgery (NOTSS) faculty. The NOTSS faculty is affiliated with the Royal College of Surgeons of Edinburgh with the mission of supporting surgical teams to advance the cognitive, social, and behavioural skills that makes surgery safe, productive, healthy, patient-centred, and team-focused. They have the greatest expertise in teaching and assessing NTS and therefore were best placed to rate video segments. Following consent and provision of demographic data, raters were randomly assigned to one of four groups of 10 videos, with each group independently rated by three raters. Ratings were performed using the validated NOTSS rating tool, which uses a 4-point scale to rate multiple elements within situation awareness and decision-making (*Table 1*)^21,22^. A ‘not observable’ option was available if a rater deemed an element absent from the video segment provided. Ratings were completed using the Research Electronic Data Capture (REDCap) platform^23^. All raters were provided with study-specific training which included a video introduction, written guidance on using the NOTSS rating tool for operative video, and in-study follow-up.

**Table 1:** Cognitive NOTSS taxonomy used for video ratings

NOTSS domain	Element
Decision-making	Considering options
	Selecting and communicating decisions
	Implementing and reviewing decisions
Situation awareness	Gathering information
	Understanding information
	Projecting and anticipating future states

To control for technical skills, video segments were also rated using the Global Operative Assessment of Laparoscopic Skills (GOALS) tool (*Supplementary Methods* and *Table S2*)^24^. A separate set of raters, recruited via the Faculty of Surgical Trainers (Royal College of Surgeons of Edinburgh) and the Technology-Enhanced Surgical Training Consensus Group (an international panel of surgical educators and researchers with an interest in data-driven surgery)^8^, assessed each video independently in parallel to the NTS group. The format of recruitment and video allocation were the same as for NTS ratings.

### Video annotation

**Fig. 1:**
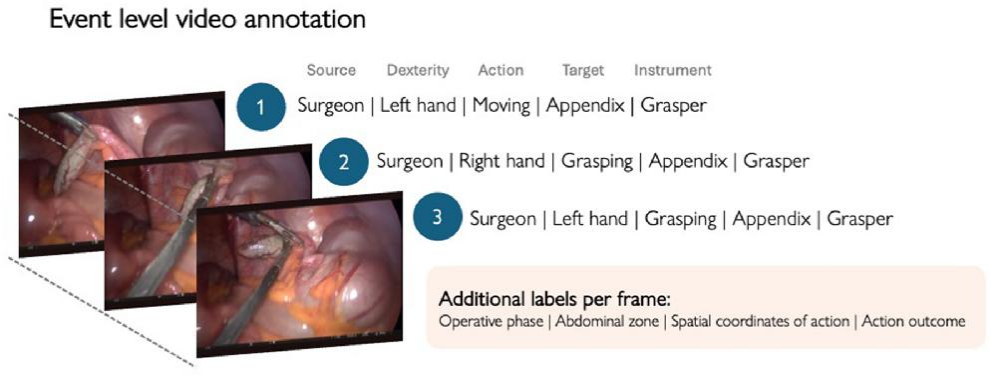
An overview of the video annotation pipeline

Full-length video annotation was performed by a single annotator (L.D.) who is clinically trained in laparoscopic appendectomy and experienced in video annotation (*Fig. 1*). Annotation was performed using Dartfish Pro S (Fribourg, Switzerland; version 11.4), a sports-analytics platform previously used successfully for operative video analysis^25^. A structured codebook was developed following established recommendations to guide annotations and maintain consistency (*Supplementary material*)^26^. Each discrete surgical event, defined as an action by the surgeon or the assistant, was temporally annotated for each gesture category (dexterity, action type, target anatomy, and instrument)^27^ and operative phase according to a previously published cognitive task analysis (*Table S3*)^28^. Spatial features were captured through *x* and *y* coordinate mapping of each event using the built-in feature within Dartfish. For example, the point of grasping the appendix was identified and the coordinates generated by the platform.

### Statistical analysis

The mean NOTSS score across the three raters was used as the summary cognitive NTS rating for each video, with a maximum score of eight. The individual rater scores were determined by the sum of situation awareness and decision-making, which in turn were determined by the mean scoring across the three elements rated for each domain. Details of how summary technical ratings were derived is available in the *Supplementary material*. For descriptive statistics, the mean (s.d.) or median (i.q.r.) across all videos were used with videos classified as high, middle and low performing based on quartiles of cognitive NTS rating.

Interrater reliability (IRR) was assessed using an ICC two-way mixed effects model^29^ and intergroup variation using a one-way analysis of variance (ANOVA). Given raters were nested within groups of 10 videos, a linear mixed-effects model was used, with group assigned as a fixed effect, and video and rater assigned as random effects to further explore rating reliability. A similar analytical approach has been used where operative videos were rated by multiple independent groups of raters^30^.

To identify predictors of cognitive NTS, a multivariable linear regression model was derived using unadjusted temporal and spatial annotations. Additional composite predictors, such as a dexterity index representing the mean number of events before dexterity change, were created from unadjusted annotations (*Supplementary Methods*). The goal was to develop a parsimonious model with the greatest explainability for the outcome variable (that is non-technical performance). Candidate predictors were selected based on univariable analysis and clinical significance. Variance inflation factor (VIF) was used to assess for collinearity between candidate predictors, with a value greater than 5 considered to be significant^31^. Selection into the final model for correlated predictors was guided by clinical significance. *P* < 0.05 was considered statistically significant, and all analyses were performed in RStudio (Version 2024.04.2+764).

## Results

### Data set characteristics

Across the 40 videos, 10 385 events were labelled, generating 87 374 data points. The mean number of events per video was 259.6 (s.d. 76.3) and the median full-length video duration (that is prior to 5-min segment editing) was 23.3 min (i.q.r. 20.6–25.2). There was no difference comparing videos by quartile for total duration and number of total and surgeon events (*Table 2*). All videos were grade 1 on the appendicitis severity scale, with 100% agreement between the two raters. Although the focus was on surgical gesture annotation, the developed codebook included label categories for 12 variables (*Table S4*).

**Table 2:** Characteristics of the data set, descriptive annotations, and non-technical ratings

	Non-technical skill group			
Variable	Lower	Middle	Upper	*P*
Number of videos	10	21	9	
Median video duration (s)	1443 (1292–1496)	1398 (1251–1554)	1306 (1187–1403)	0.49
Mean number of events	260.1 (86.6)	269.2 (71.8)	236.8 (78.6)	0.58
Mean number of surgeon events	227.9 (82.2)	231.8 (56.7)	210.3 (73.5)	0.73
Mean number of assistant events	32.2 (16.3)	36.4 (26.2)	29.5 (18.8)	0.71
**Mean non-technical rating**				
Situation awareness	2.3 (0.3)	2.8 (0.1)	3.3 (0.4)	
Decision-making	2.4 (0.4)	2.8 (0.1)	3.3 (0.3)	
Overall	4.7 (0.6)	5.6 (0.2)	6.6 (0.6)	

Of the 12 NTS video-raters, the majority were male (*n* = 11, 92%) and practised in the UK (*n* = 11, 92%). The mean number of years clinical experience of the rating panel was 22.5 years. The overall mean cognitive NTS rating was 5.6/8.0 (s.d. 0.8), with decision-making and situation awareness highly correlated (*r* = 0.8, *P*  *<* 0.001). The overall mean technical skill rating was 3.2/5.0 (s.d. 0.57). No statistically significant differences were observed in mean group-level ratings for either NTS (*P*  *=* 0.68) (*Fig. S1*) or technical skills (*P*  *=* 0.65). Furthermore, the correlation between the two constructs was not statistically significant (*r* = 0.29, *P*  *=* 0.07). IRR varied among rater groups (range: −0.48 to 0.65), with the greatest variability observed in evaluations of situation awareness, consistent with the inherently inferential nature of this category (*Tables S5*, *S6*). The linear mixed-effect model identified a cognitive NTS rater variance of 39.9%. To mitigate rater-dependent variability, ratings were aggregated across three independent raters for all downstream analyses, a commonly used approach to stabilize estimates in subjective performance assessment.

### Model derivation

**Fig. 2:**
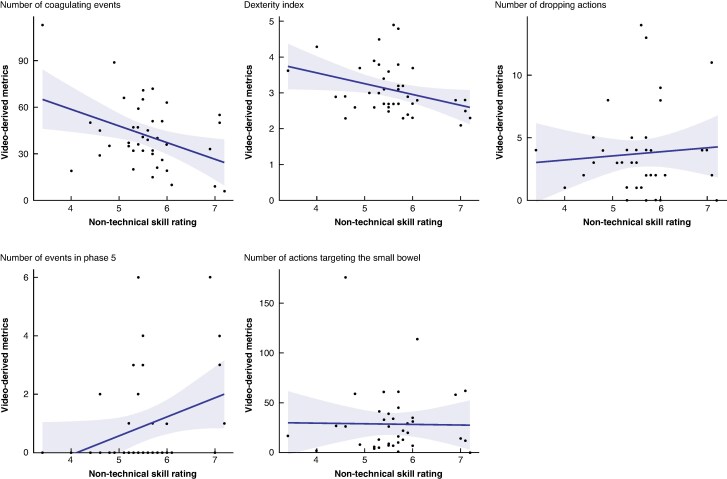
The relationship between non-technical ratings and model variables

On univariable analysis, nine variables were identified as independent predictors of NTS (*Table S7*). There was collinearity between several variables (VIF > 5), usually due to representing the same construct. For example, coagulating actions were highly correlated with use of a bipolar instrument (*r* = 0.53, *P*  *<* 0.001). After refinement, the most parsimonious model for predicting NTS was derived (*Table 3*), comprising: the number of events during phase 5, dexterity index, the number of coagulating actions, the number of dropping actions, and the number of actions targeting the small bowel (*Fig. 2*). The adjusted *R*^2^ of the final model was 0.3963.

**Table 3:** The most parsimonious model with greatest explainability for variables predictive of non-technical performance

Variable	Adjusted *R*^2^	95% c.i.	*P*
Overall model	0.3963		**<0.001**
	**Estimate**		
Number of events in phase 5	0.16	0.036 to 0.284	**0.01**
Dexterity index	−0.492	−0.803 to −0.18	**0.003**
***Number of actions***			
Coagulating	−0.018	−0.028 to −0.008	**<0.001**
Dropping	0.077	0.013 to 0.142	**0.02**
***Action target***			
Small bowel	−0.008	−0.014 to −0.001	**0.002**

## Discussion

This study explored whether surgeons’ NTS could be inferred from video-derived insights from the surgical field alone, as interpreted by expert raters blinded to wider contextual information. The findings demonstrate that specific metrics, for example the number of actions within defined operative phases and select action types, exhibit significant relationships with expert-rated NOTSS scores for decision-making and situation awareness. Although impact on clinical outcomes was not the aim of the present study, NOTSS ratings have previously been associated with patient outcomes, suggesting that the identified video-derived indicators may have meaningful implications for surgical performance. As surgical data science evolves, it is crucial to develop tools which provide meaningful insights for surgeons. By discovering video-based indicators reflective of cognitive performance, the results of this study can inform future research with the ultimate objective of developing advanced techniques to appraise skills and enhance feedback.

The variables included in the final model represent a broad range of constructs, reflecting the complexities of NTS in the OR. Prior work exploring whole OR video analysis of NTS derived models with explanatory power up to 0.335^9^. The present study had a comparable power (0.3963), reinforcing the intricacies of evaluating NTS. The majority of studies assessing NTS in the OR rely on direct observation as ground truth^32^; however, validity of video-based assessment has also been demonstrated^22^. These approaches present challenges in scalability, standardization and data availability. The present study should be interpreted as identifying video-derived technical cues that experts use as proxies for cognitive performance, rather than as a definitive measure of NTS. The weak, non-significant correlation between GOALS and NOTSS may be a result of the limited case complexity and short, edited video segments, reducing variation in technical skill while still capturing differences in cognitive performance.

Negative relationships were demonstrated between NOTSS ratings and dexterity index, the number of coagulation actions, and the number of actions targeting the small bowel. Dexterity is traditionally considered a technical skill and is included in several validated tools (for example GOALS)^24^. As a performance metric, the percentage of bimanual movements can accurately distinguish between higher and lower technical rated surgeons^33^. The findings in this study support that frequent changes in dexterity can reflect cognition, with higher-performing surgeons recognizing the need to frequently utilize non-dominant actions, a trait often less intuitive for those lacking experience. Coagulating actions were also found to be significant to non-technical performance. Accurate decision-making regarding the appropriate volume of tissue to coagulate and awareness of the proximity of surrounding structures likely results in more judicious use of this action type. This is reinforced in the recent finding that coagulating actions influence functional outcomes following surgery in select patients^13^. Small bowel–focused actions were also identified as a contributor to NTS, albeit with marginal effects. Nevertheless, awareness of small bowel positioning during setup of the surgical field can improve efficiency and flow of the operation.

A significant positive relationship was observed between dropping actions and non-technical ratings; a counterintuitive result. Acknowledging that even in the most skilled hands, errors can occur, this finding could reflect that response to, and recovery from, an undesirable event provides greater insights to surgical NTS than the absence of such actions. Further investigation in data sets with greater contextual information (for example audio) would provide further insights into the relationship between errors and cognitive performance. Increased activity during the last phase of the operation demonstrates the final opportunity to recognize and rectify potential complications (for example bleeding), increasing NTS appraisal.

The present study identifies key video-derived operative metrics relevant to non-technical performance assessment. In the short term, the most feasible application is to support formative appraisal, for example through self-reflection or video-based review. Longer term, the next phases require development of automated analytic tools, demonstration of validity, and linking to clinical outcomes. Automated annotation of gestures from operative video has already been demonstrated, notably for coagulating actions, with high specificity^34^. However, automation of some variables highlighted in this study are more challenging. Classifying dropping actions requires not just sensitivity in recognizing an ungrasped target, but specificity for the underlying principle trying to be measured (that is a technical error). These challenges could be mitigated by combining surgical field video analysis with automated NTS evaluation from video captured of the entire OR^9^, large-language model-based assessment of NTS (for example from OR transcripts)^35^, and sensors to measure physiological proxies of NTS^36^. By incorporating these automated assessments with existing performance metrics (for example kinematic data from robotics platforms)^37^, surgeons could receive feedback based on multimodal data, enhancing insights and identification of development needs. Real-time applications (for example warning highlighting excessive coagulation) could maintain patient safety and improve outcomes^13^.

The data set developed lacked audio and video of the whole OR team to complement the laparoscopic video. Although it remains unclear what the minimum signal is required to reliably measure cognitive NTS, the addition of these data streams may have reduced some rater variance and improved reliability by providing clarity of rationale from the operating surgeon’s perspective. Identifying specific attributes which influence situation awareness and decision-making ratings may also explain the observed differences in reliability. Regardless, these findings highlight the challenges of existing assessment methods, reinforce the need for objective insights, and provide foci for future research. Without the usual contextual features for NTS assessment (for example view of the OR setting, interaction between OR team members), raters may have relied more heavily on technical attributes such as tissue handling. Although mitigated through purposeful selection of raters with the necessary training and experience in using the NOTSS rating tool, the possibility remains that NTS ratings were influenced by technical features. However, this also represents a strength of the study: it provides insight into how raters extract and interpret non-technical cues in constrained or decontextualized settings, an increasingly relevant scenario for future virtual or AI-assisted performance evaluation. Finally, uncomplicated laparoscopic appendectomy may not fully stress NTS, and publicly shared videos may preferentially represent favourable performance. However, it is likely that the metrics identified become more pronounced during a complex procedure. The next logical study is to explore video-derived metrics in surgical data sets with greater diversity (for example open inclusion criteria for severity) and procedures where NTS are more critical (for example cholecystectomy)^38^.

The current study provides the first empirical evidence that cognitive NTS can be inferred from gestures captured through video of the surgical field alone when broader contextual cues are unavailable. As part of a multiphase programme, future work should focus on refining these models, expanding validation across more complex procedures and varied operative contexts, and incorporating complementary data sources. Integrating such insights into routine surgical practice via automated tools could enhance training, strengthen feedback, and ultimately improve patient outcomes.

## Collaborators

Mohamed Abdulmajed (Liverpool), Kamal Aryal (James Paget Hospital, Norwich), Ajay P. Belgaumkar (Surrey and Sussex Healthcare Trust, Redhill), Daniel Beral (Doncaster and Bassetlaw Teaching Hospitals, Doncaster), Paul M. Brennan (Centre for Clinical Brain Sciences, University of Edinburgh), Euan Bright (Royal Infirmary of Edinburgh, Edinburgh), Leo R Brown (Clinical Surgery University of Edinburgh, Edinburgh), Stuart Clark (Manchester University NHS Foundation Trust, Manchester), Sara Downey (Association of Breast Surgeons, Norfolk), Jeremy French (Freeman Hospital, Newcastle), Ewen A. Griffiths (University Hospitals Birmingham NHS Foundation Trust and University of Birmingham, Birmingham), Leo McCormick Matthews (University Hospitals Dorset, UK), M.J. Proctor (Victoria Hospital Kirkcaldy, UK), Ahmed Saad (James Cook University Hospital, Yarm), Lasitha B. Samarakoon (University Hospitals of Leicester NHS Trust, Leicester), Tim Stansfield (Leeds Teaching Hospitals NHS Trust, Leeds), Graham Sunderland (NHS Greater Glasgow and Clyde, Glasgow, UK), John V. Taylor (University Hospital Aintree, Liverpool), James Tomlinson (Sheffield Teaching Hospitals, Sheffield, UK), John Wayman (North Cumbria Integrated Care, Carlisle), J. A. Young (Borders General Hospital, Melrose)

## Supplementary Material

znag015_Supplementary_Data

## Data Availability

The data in this study are not publicly available but may be provided upon reasonable request.

## References

[1] Nepogodiev D, Martin J, Biccard B, Makupe A, Bhangu A, Nepogodiev D et al Global burden of postoperative death. Lancet 2019;393:401 30722955 10.1016/S0140-6736(18)33139-8

[2] Yule S, Flin R, Paterson-Brown S, Maran N. Non-technical skills for surgeons in the operating room: a review of the literature. Surgery 2006;139:140–149 16455321 10.1016/j.surg.2005.06.017

[3] Norton J, Janda AM, Howie E, Pohl N, Abahuje E, Harrington SD et al Impact of surgical non-technical skills on clinical outcomes: systematic review. Br J Surg 2025;113:znaf271 41459916 10.1093/bjs/znaf271 PMC12746291

[4] Parmar KL, Law J, Carter B, Hewitt J, Boyle JM, Casey P et al Frailty in older patients undergoing emergency laparotomy: results from the UK observational Emergency Laparotomy and Frailty (ELF) study. Ann Surg 2021;273:709–718 31188201 10.1097/SLA.0000000000003402

[5] Suliburk JW, Buck QM, Pirko CJ, Massarweh NN, Barshes NR, Singh H et al Analysis of human performance deficiencies associated with surgical adverse events. JAMA Netw Open 2019;2:e198067 31365107 10.1001/jamanetworkopen.2019.8067 PMC6669897

[6] Dedy NJ, Fecso AB, Szasz P, Bonrath EM, Grantcharov TP. Implementation of an effective strategy for teaching nontechnical skills in the operating room: a single-blinded nonrandomized trial: a single-blinded nonrandomized trial. Ann Surg 2016;263:937–941 26079900 10.1097/SLA.0000000000001297

[7] Yule S, Dearani JA, Pugh C. Surgical instant replay—a national video-based performance assessment toolbox. JAMA Surg 2023;158:1344–1345 37755836 10.1001/jamasurg.2023.1803 PMC13112082

[8] Dick L, Tallentire VR, Docherty AB, Smink DS, Skipworth RJE, Yule S. Data-driven metrics of operative performance: the Technology-Enhanced Surgical Training (TEST) Delphi consensus study. J Surg Educ 2025;82:103701 40972286 10.1016/j.jsurg.2025.103701

[9] Harari RE, Dias RD, Kennedy-Metz LR, Varni G, Gombolay MC, Yule S et al Deep learning analysis of surgical video recordings to assess nontechnical skills. JAMA Netw Open 2024;7:e2422520 39083274 10.1001/jamanetworkopen.2024.22520 PMC11292454

[10] Dick L, Boyle CP, Skipworth RJE, Smink DS, Tallentire VR, Yule S. Automated analysis of operative video in surgical training: scoping review. BJS Open 2024;8:zrae124 39413048 10.1093/bjsopen/zrae124 PMC11482280

[11] Olsen RG, Andersen AG, Hung AJ, Svendsen MBS, Dagnæs-Hansen JA, Konge L et al Untangling surgical gesture analysis—are we even speaking the same language? A systematic review. Surg Endosc 2025;39:1–20 10.1007/s00464-025-11907-x 40739419

[12] Nwoye CI, Yu T, Gonzalez C, Seeliger B, Mascagni P, Mutter D et al Rendezvous: attention mechanisms for the recognition of surgical action triplets in endoscopic videos. Med Image Anal 2022;78:102433 35398658 10.1016/j.media.2022.102433

[13] Heard JR, Ghaffar U, Ma R, Yang CH, Assel M, Wagner C et al Surgical performance metrics for 1-year patient-reported outcomes after radical prostatectomy. JAMA Surg 2025;160:674–680 40305032 10.1001/jamasurg.2025.0931 PMC12044537

[14] Gamborg ML, Salling LB, Rölfing JD, Jensen RD. Training technical or non-technical skills: an arbitrary distinction? A scoping review. BMC Med Educ 2024;24:1451 39696166 10.1186/s12909-024-06419-6 PMC11654166

[15] Masoomi H, Nguyen NT, Dolich MO, Mills S, Carmichael JC, Stamos MJ. Laparoscopic appendectomy trends and outcomes in the United States: data from the Nationwide Inpatient Sample (NIS), 2004–2011. Am Surg 2014;80:1074–1077 25264663

[16] Paterson HM, Qadan M, de Luca SM, Nixon SJ, Paterson-Brown S. Changing trends in surgery for acute appendicitis. Br J Surg 2008;95:363–368 17939131 10.1002/bjs.5961

[17] Lee M, Paavana T, Mazari F, Wilson TR. The morbidity of negative appendicectomy. Ann R Coll Surg Engl 2014;96:517–520 25245730 10.1308/003588414X13946184903801 PMC4473437

[18] Garst GC, Moore EE, Banerjee MN, Leopold DK, Burlew CC, Bensard DD et al Acute appendicitis: a disease severity score for the acute care surgeon. J Trauma Acute Care Surg 2013;74:32–36 23271074 10.1097/TA.0b013e318278934a

[19] Currow C, Patel K, Askari A, Rabie M, Aly M, Aker M et al Current technical surgical practice of emergency appendicectomy: a cross-sectional survey of surgical registrars in the UK. Ann R Coll Surg Engl 2020;102:606–610 32501113 10.1308/rcsann.2020.0123 PMC7538747

[20] Birkmeyer JD, Finks JF, O’Reilly A, Oerline M, Carlin AM, Nunn AR et al Surgical skill and complication rates after bariatric surgery. N Engl J Med 2013;369:1434–1442 24106936 10.1056/NEJMsa1300625

[21] Yule S, Flin R, Paterson-Brown S, Maran N, Rowley D. Development of a rating system for surgeons’ non-technical skills. Med Educ 2006;40:1098–1104 17054619 10.1111/j.1365-2929.2006.02610.x

[22] Yule S, Gupta A, Gazarian D, Geraghty A, Smink DS, Beard J et al Construct and criterion validity testing of the Non-Technical Skills for Surgeons (NOTSS) behaviour assessment tool using videos of simulated operations. Br J Surg 2018;105:719–727 29601087 10.1002/bjs.10779

[23] Harris PA, Taylor R, Thielke R, Payne J, Gonzalez N, Conde JG. Research electronic data capture (REDCap)—a metadata-driven methodology and workflow process for providing translational research informatics support. J Biomed Inform 2009;42:377–381 18929686 10.1016/j.jbi.2008.08.010 PMC2700030

[24] Vassiliou MC, Feldman LS, Andrew CG, Bergman S, Leffondré K, Stanbridge D et al A global assessment tool for evaluation of intraoperative laparoscopic skills. Am J Surg 2005;190:107–113 15972181 10.1016/j.amjsurg.2005.04.004

[25] Suzuki T, Egi H, Hattori M, Tokunaga M, Sawada H, Ohdan H. An evaluation of the endoscopic surgical skills assessment using a video analysis software program. Surg Endosc 2015;29:1804–1808 25294543 10.1007/s00464-014-3863-5

[26] Meireles OR, Rosman G, Altieri MS, Carin L, Hager G, Madani A et al SAGES consensus recommendations on an annotation framework for surgical video. Surg Endosc 2021;35:4918–4929 34231065 10.1007/s00464-021-08578-9

[27] Ma R, Ramaswamy A, Xu J, Trinh L, Kiyasseh D, Chu TN et al Surgical gestures as a method to quantify surgical performance and predict patient outcomes. NPJ Digit Med 2022;5:187 36550203 10.1038/s41746-022-00738-y PMC9780308

[28] Smink DS, Peyre SE, Soybel DI, Tavakkolizadeh A, Vernon AH, Anastakis DJ. Utilization of a cognitive task analysis for laparoscopic appendectomy to identify differentiated intraoperative teaching objectives. Am J Surg 2012;203:540–545 22325336 10.1016/j.amjsurg.2011.11.002

[29] Koo TK, Li MY. A guideline of selecting and reporting intraclass correlation coefficients for reliability research. J Chiropr Med 2016;15:155–163 27330520 10.1016/j.jcm.2016.02.012 PMC4913118

[30] Scully RE, Deal SB, Clark MJ, Yang K, Wnuk G, Smink DS et al Concordance between expert and nonexpert ratings of condensed video-based trainee operative performance assessment. J Surg Educ 2020;77:627–634 32201143 10.1016/j.jsurg.2019.12.016

[31] Lucocq J, Hamilton D, Bakhiet A, Tasnim F, Rahman J, Scollay J et al Derivation and validation of a predictive model for subtotal cholecystectomy. Surg Endosc 2024;38:6551–6559 39285041 10.1007/s00464-024-11241-8 PMC11525303

[32] McMullan RD, Urwin R, Sunderland N, Westbrook J. Observational tools that quantify nontechnical skills in the operating room: a systematic review. J Surg Res 2020;247:306–322 31706538 10.1016/j.jss.2019.10.012

[33] Yang JH, Goodman ED, Dawes AJ, Gahagan JV, Esquivel MM, Liebert CA et al Using AI and computer vision to analyze technical proficiency in robotic surgery. Surg Endosc 2023;37:3010–3017 36536082 10.1007/s00464-022-09781-y

[34] Olsen RG, Bjerrum F, Andersen AG, Konge L, Røder A, Svendsen MBS. Development of an artificial intelligence algorithm for automated surgical gestures annotation. J Robot Surg 2025;19:404 40681897 10.1007/s11701-025-02556-2 PMC12274238

[35] Obuseh M, Singh S, Anton NE, Gardiner R, Stefanidis D, Yu D. Feasibility of large language models for assessing and coaching surgeons’ non-technical skills. Npj Health Syst 2025;2:25 40678790 10.1038/s44401-025-00027-2 PMC12263418

[36] Howie EE, Ambler O, Gunn EGM, Dias RD, Wigmore SJ, Skipworth RJE et al Surgical sabermetrics: a scoping review of technology-enhanced assessment of nontechnical skills in the operating room: a scoping review of technology-enhanced assessment of non-technical skills in the operating room. Ann Surg 2024;279:973–984 38258573 10.1097/SLA.0000000000006211 PMC11086675

[37] Boal MWE, Anastasiou D, Tesfai F, Ghamrawi W, Mazomenos E, Curtis N et al Evaluation of objective tools and artificial intelligence in robotic surgery technical skills assessment: a systematic review. Br J Surg 2024;111:znad331 37951600 10.1093/bjs/znad331 PMC10771126

[38] Dick L, Boyle C, Norton J, Ruth Tallentire V, Skipworth RJE, Yule S. A pilot study of real-world operative video analysis for assessing non-technical skills in surgical trainees. J Surg Protoc Res Methodol 2026;2026:snaf022

