# Supplementary material for: Identifying novel indicators of non-technical skills derived from operative video annotation
Source: Br J Surg. 2026 Feb 19;113(3):znag015. doi: 10.1093/bjs/znag015 (PMC13036482; doi:10.1093/bjs/znag015)
Supplement: znag015_Supplementary_Data [file znag015_supplementary_data.docx]

**Identifying novel indicators of non-technical skills derived from operative video annotation**

**Supplementary Material**

Lachlan Dick MBChB ^1,2,3^, Connor Boyle MBChB ^1,3^, Victoria Ruth Tallentire MD ^2^, Joe Norton MBBS ^1,3^, Emma Howie MBChB ^1,3^, Douglas S Smink MD, MPH ^4^, Richard JE Skipworth MD ^1,3^, Steven Yule PhD ^1,3^ on behalf of the Surgical Video Grading and Assessment (SVGA) Group.

1. Surgical Sabermetrics Laboratory, Usher Institute, University of Edinburgh, EH16 4UX
2. Medical Education Directorate, Royal Infirmary of Edinburgh, NHS Lothian, EH16 4SA
3. Clinical Surgery, University of Edinburgh, EH16 4SA
4. Department of Surgery, Brigham and Women’s Hospital/Harvard Medical School, Boston, MA

**Corresponding author:** Lachlan Dick. Surgical Sabermetrics Laboratory, Usher Institute, University of Edinburgh, EH16 4UX. [lachlan.dick@ed.ac.uk](mailto:lachlan.dick@ed.ac.uk). **ORCID:** 0000-0002-4341-9501; **Twitter** @lachiedick

**Supplementary Materials - Index**

[**Supplementary Methods 2**](#_i3qsc0by16ti)

[Technical skill rating tool 2](#_5o9cevsqq9vb)

[Additional video-derived metrics 2](#_kby41e828v5e)

[Statistical analysis 3](#_g83wh03k32p2)

[**Supplementary Tables 4**](#_vl06ojllfi72)

[Table S1 4](#_n3iho9ooeddw)

[Table S2 5](#_3kdc216pgaq)

[Table S3 6](#_nuhxgdpe5tfg)

[Table S4 7](#_p3x354a1v7ag)

[Table S5 8](#_r97m0kjkmsbr)

[Table S6 8](#_h84nt55071h)

[Table S7 9](#_kswzylhwluon)

[**Supplementary Figures 11**](#_utxdqiwth0ry)

[Figure S1 11](#_7vxmapqmlgvw)

[**Annotation codebook 12**](#_ecx7rkqcwso)

[**References 18**](#_31zzs0ufkpzo)

# Supplementary Methods

## Technical skill rating tool

The Global Objective Assessment of Laparoscopic Skills (GOALS) framework was used to rate technical skills.[^1^](https://paperpile.com/c/anJjOQ/qGfQ) This tool consists of five domains to assess technical proficiency: autonomy, bimanual dexterity, depth perception, efficiency and tissue handling. Each domain is rated using a 5-point Likert scale, with domain descriptors for scores of one, three and five (supplementary table two). The GOALS tool demonstrates a high inter-rater reliability (intra-class correlation coefficient up to 0.89),[^1^](https://paperpile.com/c/anJjOQ/qGfQ) with validity demonstrated across multiple procedure types, including laparoscopic appendicectomy.[^2^](https://paperpile.com/c/anJjOQ/F0Bk) For this study, the autonomy domain was omitted from ratings. This was due to the lack of data to allow for accurate assessment, and is a practice which has been adopted in similar studies.[^3^](https://paperpile.com/c/anJjOQ/DvnW)

## Additional video-derived metrics

In addition to the metrics extracted unadjusted from video annotations, several metrics were developed based on unadjusted metrics. These were developed based on clinical significance and potential for reflecting non-technical skills (NTS).

| **Metric** | **Derived from** | **Definition** | **Rationale** |
| --- | --- | --- | --- |
| Event rate | Total video duration and number of events | The ratio of video duration to number of events | The relationship between scenario duration and NTS has previously been established.[^4^](https://paperpile.com/c/anJjOQ/Sbz8) This metric provides further nuance by considering the number of events (i.e., productivity) during the video duration. |
| Dexterity index | Unadjusted left and right dexterity | The mean number of events before a dexterity change for each video. | Simple dexterity alone may not provide insights into NTS. By focussing on the distribution of dexterity, greater insights into cognitive processes could be achieved. |
| Move ratio | Unadjusted moving and grasping actions | The ratio of moving to grasping actions. | Previous literature has identified increased grasping actions are associated with longer operative durations. By eliminating grasping actions in favour of moving actions (e.g., during mesoappendix dissection), tension can be maintained and efficiency improved.[^5^](https://paperpile.com/c/anJjOQ/4pD1) |
| Coagulate ratio | Unadjusted coagulating and dissecting actions | The ratio of coagulating to dissecting actions | Coagulating actions have previously been identified as influencing functional outcomes in select patients.[^6^](https://paperpile.com/c/anJjOQ/9r7u) Conscious use of dissecting in favour of coagulating actions may reflect enhanced decision-making and situation awareness |
| Centrality index | Unadjusted *x* and *y* coordinates for each event | An index from 0 to 1 to indicate the proximity of each event to the absolute centre of the screen (i.e., *x*=50, *y*=50). A score of 1 indicates all events are at the centre. | Tracking surgical instruments has been utilised in determining skill proficiency.[^7^](https://paperpile.com/c/anJjOQ/dRiA) This metric aims to proxy surgical movements by plotting 2-dimensional positioning. |

## Statistical analysis

As the summary technical rating for each of the 40 videos, the mean video rating across the three surgeon ratings was used. The individual surgeon ratings were determined by the mean scoring across the four assessed domains. The total score available for technical skills was 5.

#

#

#

# Supplementary Tables

## Table S1

The appendicitis severity grading used.[^8^](https://paperpile.com/c/anJjOQ/6AIe)

| **Grade** | **Definition** |
| --- | --- |
| 1 | Inflamed appendix |
| 2 | Gangrenous appendix |
| 3 | Perforated appendix - free fluid |
| 4 | Perforated appendix - localised abscess |
| 5 | Perforated appendix - generalised peritonitis |

## Table S2

GOALS domains and descriptors used in this study.

|  | **1** | **2** | **3** | **4** | **5** |
| --- | --- | --- | --- | --- | --- |
| Bimanual dexterity | Uses only one hand, ignores non-dominant hand, poor coordination between hands) |  | Uses both hands, but does not optimise interaction between hands |  | Expertly uses both hands in a complimentary manner to provide optimal exposure |
| Depth perception | Constantly overshoots target, wide swings, slow to correct |  | Some overshooting or missing of target, but quick to correct |  | Accurately directs instruments in the correct plane to target |
| Efficiency | Uncertain, inefficient efforts; many tentative movements; constantly changing focus or persisting without progress |  | Slow, but planned movements are reasonably organised |  | Confident, efficient and safe conduct, maintains focus on task until it is better performed by way of an alternative approach |
| Tissue handling | Rough movements, tears tissue, injures adjacent structures, poor grasper control, grasper frequently slips |  | Handles tissues reasonably well, minor trauma to adjacent tissue (ie, occasional unnecessary bleeding or slipping of the grasper |  | Handles tissues well, applies appropriate traction, negligible injury to adjacent structures |

## Table S3

Phase definition used for laparoscopic appendectomy.[^9^](https://paperpile.com/c/anJjOQ/On9k)

| **Phase of the operation** | **Description** | **Label** |
| --- | --- | --- |
| Peritoneal access | Places umbilical, suprapubic and left lower quadrant ports  Establishes pneumoperitoneum  Inspects abdomen and appendix | Phase 1 |
| Exposure and assessment of the appendix | Exposes appendix  Mobilisation of appendix to locate appendiceal base | Phase 2 |
| Dissection and division of the appendix | Determines anatomy of mesoappendix and appendiceal base  Divides mesoappendix  Divides appendiceal base | Phase 3 |
| Removal of the appendix and inspection of the surgical bed | Remove appendix  Inspect operative bed | Phase 4 |
| Closure | Remove ports  Close fascia and skin | Phase 5 |

## Table S4

Annotation characteristics of the developed dataset, ranked in descending order of number of datapoints.

| **Annotation category** | **Number of datapoints** |
| --- | --- |
| Source | 10385 |
| Operative phase | 10385 |
| Abdominal zone of event | 10385 |
| Action | 9038 |
| Instrument | 9038 |
| Dexterity | 8945 |
| Target | 8943 |
| Spatial coordinates | 8717 |
| Camera event | 1347 |
| Grasp position for appendix | 982 |
| Unsuccessful action | 273 |
| Unsuccessful action with negative outcome (e.g., bleeding) | 10 |
|  | **Number of unique labels** |
| Action type | 13 |
| Target* | 31 |
| Instrument | 13 |

* includes target anatomy (e.g., appendix), anatomical regions (e.g., right iliac fossa) and artificial structures (e.g., drain)

## Table S5

The IRR for technical (S5) and non-technical (S6) ratings. Individual domain IRR are reported for non-technical ratings.

**Technical skills**

| **Group** | **ICC** | **95% CI** | **p-value** |
| --- | --- | --- | --- |
| A | 0.7 | 0.13-0.9 | 0.01 |
| B | 0.21 | -1 - 0.77 | 0.31 |
| C | 0.64 | -0.04 - 0.9 | 0.03 |
| D | 0.12 | -1.58 - 0.78 | 0.35 |

## Table S6

**Non-technical skills**

| **Group** | **ICC** | **95% CI** | **p-value** |
| --- | --- | --- | --- |
| A | 0.35 | -0.29 - 0.78 | 0.142 |
| Situation awareness | 0.13 | -0.37 - 0.65 | 0.303 |
| Decision-making | 0.53 | -0.16 - 0.86 | 0.06 |
| B | 0.65 | 0.05 - 0.9 | 0.02 |
| Situation awareness | 0.64 | 0.009 - 0.9 | 0.02 |
| Decision-making | 0.62 | 0.001 - 0.89 | 0.02 |
| C | -0.48 | -0.76 - 0.19 | 0.94 |
| Situation awareness | -0.46 | -0.76 - 0.22 | 0.93 |
| Decision-making | -0.5 | -0.78 | 0.94 |
| D | 0.12 | -1.62 | 0.34 |
| Situation awareness | -0.49 | -2.44 - 0.7 | 0.78 |
| Decision-making | 0.66 | -0.12 - 0.95 | 0.04 |

##

##

##

##

##

## Table S7

Univariable linear regression analysis for each metric. Green highlighted cells represent metrics independently predictive of cognitive NTS.

| **Metric** | **R2** | **Lower CI** | **Upper CI** | **p-value** |
| --- | --- | --- | --- | --- |
| Video duration | 0.013 | -0.001 | 0.0005 | 0.484 |
| Total number of events | 0.008 | -0.004 | 0.002 | 0.59 |
| Event rate | 0.0007 | -0.184 | 0.216 | 0.871 |
| Number of surgeon events | 0.007 | -0.005 | 0.003 | 0.605 |
| Number of assistant events | 0.003 | -0.014 | 0.01 | 0.754 |
| **Number of camera events:** |  |  |  |  |
| Removed from abdomen | 0.0003 | -0.069 | 0.077 | 0.912 |
| Inserted into the abdomen | 0.003 | -0.081 | 0.073 | 0.912 |
| Camera cleaned | 0.005 | -0.01 | 0.064 | 0.656 |
| View of the port | 0.002 | -0.018 | 0.013 | 0.76 |
| **Number of events within each phase:** |  |  |  |  |
| Phase 1 | 0 | -0.014 | 0.014 | 0.96 |
| Phase 2 | 0.019 | -0.004 | 0.01 | 0.392 |
| Phase 3 | 0.017 | -0.007 | 0.003 | 0.417 |
| Phase 4 | 0.011 | -0.007 | 0.003 | 0.516 |
| Phase 5 | 0.097 | -0.0006 | 0.298 | 0.05 |
| **Dexterity:** |  |  |  |  |
| Number of left-hand events | 0.0005 | -0.009 | 0.008 | 0.888 |
| Number of right-hand events | 0.018 | -0.009 | 0.004 | 0.403 |
| Dexterity ratio | 0.022 | -1.07 | 0.4 | 0.362 |
| Dexterity index | 0.115 | -0.734 | -0.033 | 0.03 |
| **Number of actions:** |  |  |  |  |
| Aspirating | 0.003 | -0.025 | 0.035 | 0.742 |
| Clipping | 0.005 | -0.329 | 0.512 | 0.662 |
| Coagulating | 0.151 | -0.025 | -0.003 | 0.01 |
| Cutting | 0.0002 | -0.05 | 0.05 | 0.922 |
| Dissecting | 0.0006 | -0.015 | 0.018 | 0.876 |
| Dropping | 0.007 | -0.058 | 0.097 | 0.615 |
| Grasping | 0.008 | -0.012 | 0.591 | 0.591 |
| Inserting | 0.012 | -0.208 | 0.424 | 0.493 |
| Irrigating | 0 | -0.054 | 0.055 | 0.98 |
| Moving | 0.011 | -0.006 | 0.012 | 0.518 |
| Removing | 0.084 | -0.025 | 0.614 | 0.07 |
| Suturing | 0.097 | -0.0002 | 1.046 | 0.05 |
| Tying | 0.043 | -0.034 | 0.157 | 0.199 |
| Move ratio | 0.047 | -0.066 | 0.339 | 0.181 |
| Coagulate ratio | 0.019 | -0.016 | 0.037 | 0.42 |
| Dissect ratio | 0.11 | 0.003 | 0.718 | 0.05 |
| **Number of actions targeting:** |  |  |  |  |
| Appendix | 0.003 | -0.011 | 0.008 | 0.747 |
| Mesoappendix | 0.097 | -0.02 | 0 | 0.05 |
| Other structures | 0.013 | -0.005 | 0.01 | 0.486 |
| Small bowel | 0.0002 | -0.008 | 0.007 | 0.931 |
| **Number of actions using:** |  |  |  |  |
| Bipolar | 0.249 | -0.018 | -0.005 | 0.001 |
| Clip applicator | 0.004 | -0.297 | 0.441 | 0.696 |
| Grasper | 0.002 | -0.004 | 0.006 | 0.743 |
| Hook diathermy | 0 | -0.01 | 0.01 | 0.989 |
| Knot pusher | 0.065 | -0.03 | 0.274 | 0.112 |
| Maryland | 0.006 | -0.113 | 0.07 | 0.644 |
| Needle holder | 0.097 | 0 | 0.08 | 0.05 |
| Scissors | 0.0002 | -0.025 | 0.023 | 0.929 |
| Suction | 0.009 | -0.008 | 0.015 | 0.56 |
| Trocar | 0.097 | -0.0002 | 0.485 | 0.05 |
| Surgical ties | 0.007 | -0.149 | 0.089 | 0.609 |
| Suture | 0.001 | -1.309 | 1.057 | 0.83 |
| Vessel sealing device | 0.063 | -0.003 | 0.028 | 0.117 |
| Centrality index | 0.005 | -8.263 | 5.207 | 0.649 |

#

#

#

#

# Supplementary Figures

## Figure S1

Distribution of cognitive NTS ratings by video group.


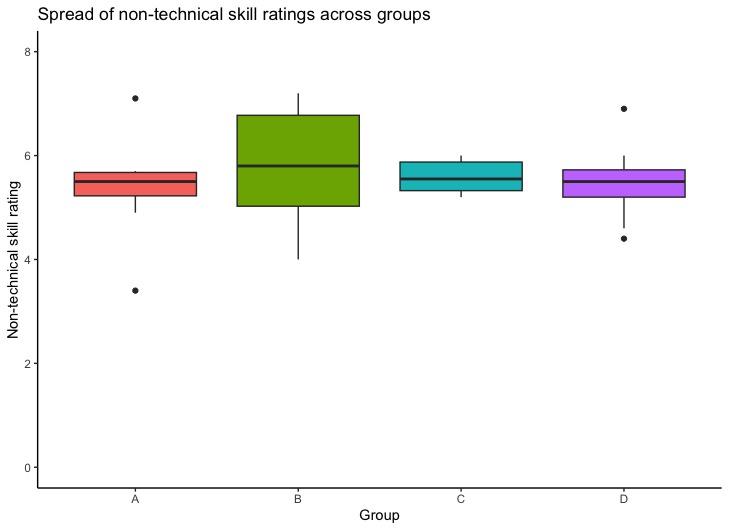


##

#

# Annotation codebook

**Temporal annotations**

**Operative phases**

Defined from a previous cognitive task analysis on the phases, steps and decision points during laparoscopic appendicectomy.[^9^](https://paperpile.com/c/anJjOQ/On9k) Originally, six phases are described, however; the first phase refers to events occurring before the laparoscopic portion of the operation and is therefore removed from this codebook.

| Phase of the operation | Description | Label |
| --- | --- | --- |
| Peritoneal access | Places umbilical, suprapubic and left lower quadrant ports  Establishes pneumoperitoneum  Inspects abdomen and appendix | Phase 1 |
| Exposure and assessment of the appendix | Exposes appendix  Mobilisation of appendix to locate appendiceal base | Phase 2 |
| Dissection and division of the appendix | Determines anatomy of mesoappendix and appendiceal base  Divides mesoappendix  Divides appendiceal base | Phase 3 |
| Removal of the appendix and inspection of the surgical bed | Remove appendix  Inspect operative bed | Phase 4 |
| Closure | Remove ports  Close fascia and skin | Phase 5 |

**Source**

| Dexterity | Description |
| --- | --- |
| Surgeon | The event is attributable to the surgeon. |
| Assistant | The event is attributable to the assistant (e.g., camera event). |

**Dexterity**

| Dexterity | Description |
| --- | --- |
| Left | Event uses the left hand. |
| Right | Event uses the right hand. |

**Action**

| Action | Description |
| --- | --- |
| Aspirates | Aspiration of fluid from abdomen (e.g. blood, saline) |
| Clips | Applies a synthetic clip to a structure (e.g. appendiceal artery) |
| Coagulates | Use of any electrocautery device to coagulate tissue |
| Cuts | Use of any instrument to perform sharp dissection |
| Dissects (bluntly) | Use of any instrument to perform blunt dissection |
| Drops | Inadvertently dropping a structure (e.g. appendix, small bowel, suture material) |
| Grasps | Use of any instrument to grasp a structure (e.g. appendix, small bowel, suture material) |
| Irrigates | Washout with fluid |
| Moves | Use of any instrument to move a structure (e.g. appendix, small bowel, suture material) |
| Passes | Deliberate passing of a structure from one hand to another |
| Sutures | Uses suture material to place stitch |
| Staples | Use of a stapling device |
| Ties | Use of a suture material to ligate a structure (e.g. base of appendix, appendicular artery) |

**Target**

| Target | Notes |
| --- | --- |
| **Anatomical** |  |
| Adhesion | Any connective tissue that is not part of embryological development |
| Appendix | Any part of the vermiform appendix (excluding base - see below) |
| Appendicular artery | Any vascular structure identified within the mesoappendix |
| Base of appendix | The junction between the appendix and caecum. After division of the appendix, the remaining portion of the appendix (‘the stump’) is classified as base. |
| Bladder | Any part of the peritonised bladder |
| Caecum | Any part of the caecum |
| Fluid | Blood, irrigation fluid |
| Ileal fat pad | Mesentery associated with the terminal ileum |
| Mesoappendix | The mesentery associated with the appendix |
| Omentum | Any part of the omentum |
| Ovary (left) | Any part of the left ovary or fallopian tube |
| Ovary (right) | Any part of the right ovary of fallopian |
| Peritoneum | Any part of the parietal peritoneum |
| Sigmoid colon | Any part of the sigmoid colon |
| Small bowel (general) | Any part of the small bowel (excluding terminal ileum - see below) |
| Terminal ileum | Any part of the small bowel directly proximal to the ileocaecal junction. For simplicity, this is any part of the small bowel, proximal to the ileocaecal junction and within the field of view. |
| Uterus | Any part of the uterus |
| **Non-anatomical** |  |
| Bag | Any synthetic bag material |
| Drain | Any synthetic drain material |
| Needle | Any intracorporeal or extracorporeal needle |
| Suture | Any synthetic material |
| Swab | Any synthetic swab material |
| Trocar | Any device used to facilitate access into the abdominal cavity |

**Instrument**

| Instrument | Description |
| --- | --- |
| Bipolar device | Any device which passes current between two electrodes to achieve haemostasis. This does not include grasper devices with monopolar attached (see below) or vessel sealing devices (see below). |
| Clip applicator | Metal clip or Haem-o-lock |
| Endoloop | Pre-made or self-tied |
| Grasper | Any type |
| Hook | Any type |
| Knot pusher | Any type |
| Needle holder | Any type |
| Scissors | Any type |
| Suction | Any type |
| Suture | Any suture material |
| Swab | Any synthetic swab material |
| Vessel sealing device | Harmonic, Thunderbeat |

#

**Zone**

| Zone | Description |
| --- | --- |
| Supracolic compartment | Superior to the transverse mesocolon |
| Infracolic compartment | Inferior to the transverse mesocolon |
| Pelvis | Below the level of the sacral promontory |

##

**Camera event**

| Camera events | Description |
| --- | --- |
| Removed from abdomen | Camera removed from abdominal cavity (e.g. for cleaning) |
| Port view | During a surgical sequence, the view is from within the port (i.e. not providing a full view of target anatomy) |
| Obscured view | Camera view obscured (e.g. by blood, fog, tissue) |
| Optics change | Change of view by rotating optics (30^o^ camera only) |

**Spatial annotations**

**Position**

x + y coordinates of each event.

| x + y coordinate | Description |
| --- | --- |
| Position of event | x + y coordinate of event |

**Outcome**

| Outcome | Description |
| --- | --- |
| Successful | Event results in the intended outcome |
| Unsuccessful | Event does not result in the intended outcome |
| Unsuccessful + undesirable outcome | Event does not result in the intended outcome + has an undesirable event (e.g. event results in bleeding). |

##

# References

1. [Vassiliou MC, Feldman LS, Andrew CG, et al. A global assessment tool for evaluation of intraoperative laparoscopic skills. *Am J Surg*. 2005;190:107–113.](http://paperpile.com/b/anJjOQ/qGfQ)

2. [Gumbs AA, Hogle NJ, Fowler DL. Evaluation of resident laparoscopic performance using global operative assessment of laparoscopic skills. *J Am Coll Surg*. 2007;204:308–313.](http://paperpile.com/b/anJjOQ/F0Bk)

3. [Jin A, Yeung S, Jopling J, et al. Tool detection and operative skill assessment in surgical videos using region-based convolutional neural networks. In: 2018 IEEE Winter Conference on Applications of Computer Vision (WACV). IEEE; 2018:691–699.](http://paperpile.com/b/anJjOQ/DvnW)

4. [Doumouras AG, Hamidi M, Lung K, et al. Non-technical skills of surgeons and anaesthetists in simulated operating theatre crises: Non-technical skills during operative crises. *Br J Surg*. 2017;104:1028–1036.](http://paperpile.com/b/anJjOQ/Sbz8)

5. [Sanmoto Y, Kawami A, Goto Y, et al. Correlation between forceps grasp count and skill proficiency in single-incision laparoscopic percutaneous extraperitoneal closure: A retrospective single-center study. *Asian J Endosc Surg*. 2025;18:e13403.](http://paperpile.com/b/anJjOQ/4pD1)

6. [Heard JR, Ghaffar U, Ma R, et al. Surgical performance metrics for 1-year patient-reported outcomes after radical prostatectomy. *JAMA Surg*. 2025;160:674–680.](http://paperpile.com/b/anJjOQ/9r7u)

7. [Dick L, Boyle CP, Skipworth RJE, et al. Automated analysis of operative video in surgical training: scoping review. *BJS Open*. 2024;8:zrae124.](http://paperpile.com/b/anJjOQ/dRiA)

8. [Garst GC, Moore EE, Banerjee MN, et al. Acute appendicitis: a disease severity score for the acute care surgeon. *J Trauma Acute Care Surg*. 2013;74:32–36.](http://paperpile.com/b/anJjOQ/6AIe)

9. [Smink DS, Peyre SE, Soybel DI, et al. Utilization of a cognitive task analysis for laparoscopic appendectomy to identify differentiated intraoperative teaching objectives. *Am J Surg*. 2012;203:540–545.](http://paperpile.com/b/anJjOQ/On9k)
